# Deep Learning Technology and Image Sensing

**DOI:** 10.3390/s24165130

**Published:** 2024-08-08

**Authors:** Suk-Ho Lee, Dae-Ki Kang

**Affiliations:** Department Computer Engineering, Dongseo University, Busan 47011, Republic of Korea; dkkang@dongseo.ac.kr

The scientific landscape is constantly evolving, marked by groundbreaking advancements in imaging, sensing, and machine learning that expand the realms of possibility across various disciplines. Today, deep learning-based computing technology plays a pivotal role in enhancing the quality and reliability of image recognition data. For instance, in the realm of autonomous driving, deep learning-based fusion of data from front camera sensors and radars enables the significant improvement of sensor performance. Additionally, deep learning-driven computer vision technologies contribute to enhancing smartphone camera applications, enabling functionalities such as face recognition, panorama photography, depth/geometry detection, and high-quality magnification and detection. This Special Issue, entitled “Deep Learning Technology and Image Sensing”, encompasses all topics related to applications utilizing deep learning-based image and video sensing technologies. In this editorial overview, we present eleven papers that are published in this Special Issue that demonstrate innovative approaches and methodologies spanning diverse domains. We divide the domains into applications across medical imaging and healthcare, image enhancement, object detection, and innovation in image sensing technologies.

Several papers in the domain of medical imaging and healthcare applications focus on medical imaging and its applications in healthcare. From liver segmentation and brain tumor classification to the automated detection of optic cup and disc edges in glaucoma patients, these studies demonstrate the power of deep learning frameworks in improving the diagnostic accuracy and clinical decision-making.

Liver segmentation in MRI images poses challenges due to the variable characteristics and lack of HoU-based preprocessing. To tackle this problem, in [1], the authors explore state-of-the-art segmentation networks on T1-weighted MRI scans from a public dataset. A novel cascaded network is proposed, achieving superior results with a DSC of 95.15% and IoU of 92.10% on expert-labeled liver masks. The framework demonstrates high accuracy and holds promise for medical imaging applications.

Rapid and accurate brain tumor detection is crucial for patient health. Despite recent advancements in AI methods, precise diagnoses remain challenging. The work in [2], entitled “A Novel Approach for Brain Tumor Classification Using an Ensemble of Deep and Hand-Crafted Features”, proposes a novel approach using an ensemble of hand-crafted features and deep features from VGG16. The ensemble improves discrimination, achieving a 99% accuracy when classified using SVM or KNN. This method demonstrates reliability for MRI-based tumor detection, offering robustness and potential real-world deployment. Validation through cross-tabulated data confirms the model’s good performance.

The study in [3], entitled “Identifying the Edges of the Optic Cup and the Optic Disc in Glaucoma Patients by Segmentation”, focuses on automating the detection of optic cup and optic disc edges in fundus images of glaucoma patients, crucial for early diagnosis. Utilizing a modified U-Net model, we evaluate the segmentation performance across multiple datasets. The postprocessing techniques utilized enhance visualization for improved cup-to-disc ratio analysis. The results demonstrate a promising segmentation efficiency, which is particularly beneficial for clinical applications.

Advancements in image enhancement techniques are evident in the papers exploring image-to-image translation in astronomy, super-resolution imaging, and depth map super-resolution. These studies leverage sophisticated algorithms to enhance the image quality, extract meaningful information, and address challenges in various imaging modalities.

In the work of [4], entitled “Hubble Meets Webb, Image-to-Image Translation in Astronomy”, the authors explore the translation of Hubble Space Telescope (HST) data into James Webb Space Telescope (JWST) imagery using various techniques. The proposed method emphasizes the importance of image registration and introduces uncertainty estimation to enhance the translation reliability. This approach aids in preparatory strategies for JWST observations, offering predictive insights when JWST data are unavailable, making it the first attempt at sensor-to-sensor image translation in astronomy.

Meanwhile, the study in [5], entitled “Kernel Estimation Using Total Variation Guided GAN for Image Super-Resolution”, addresses artifacts in image super-resolution. The authors propose a Total Variation Guided KernelGAN focusing on structural details. Their experimental results demonstrate this method’s effectiveness in accurately estimating kernels, leading to improved super-resolution algorithm performance.

Another approach for super-resolution is descibed in the study entitled “Fully Cross-Attention Transformer for Guided Depth Super-Resolution” [6], which presents a fully transformer-based network for depth map super-resolution, addressing issues in existing guided super-resolution methods. This approach utilizes a cascaded transformer module with a novel cross-attention mechanism, seamlessly guiding the color image into the depth upsampling process. Leveraging a window partitioning scheme ensures linear complexity for high-resolution images and extensive experiments demonstrate the superiority of the proposed method over state-of-the-art approaches.

Besides super-resolution, a low-light image enhancement technique has also been proposed in the work in [7], entitled “Low-Light Image Enhancement Using Hybrid Deep-Learning and Mixed-Norm Loss Functions”. The authors propose a method for enhancing low-light images using a hybrid deep learning network and mixed-norm loss functions. Their approach includes a decomposition-net to separate reflectance and illuminance, an illuminance enhance-net to improve illuminance while reducing artifacts, and a chroma-net to mitigate color distortion. YCbCr channels are utilized for training and restoration to account for RGB channel correlations. Mixed-norm loss functions enhance stability and reduce blurring by reflecting reflectance, illuminance, and chroma properties. The experimental results show significant subjective and objective improvements compared to state-of-the-art deep-learning methods.

The integration of artificial intelligence and object detection plays a pivotal role in driving innovation across different fields. The papers in this category present real-time action sensing and detection systems, weakly supervised object detection methods, and novel approaches for brain–computer interfaces. These studies underscore the importance of leveraging AI and ML techniques to automate tasks, improve efficiency, and enable the development of new applications.

The work in [8] (“A Real-Time Subway Driver Action Sensing and Detection Based on Lightweight ShuffleNetV2 Network”) proposes a lightweight two-stage model for the real-time monitoring of subway drivers’ actions using surveillance cameras. Ensuring subway train safety relies heavily on the actions of drivers. To this end, the model utilizes MobileNetV2-SSDLite for driver detection and an enhanced ShuffleNetV2 network for action recognition, achieving a superior performance over existing models and meeting runtime requirements for practical deployment.

Meanwhile, the authors of [9] (“Instance-Level Contrastive Learning for Weakly Supervised Object Detection”) propose instance-level contrastive learning (ICL) in order to mine reliable instance representations from images via contrastive loss.They introduce instance-diverse memory updating (IMU) to capture diverse instances and memory-aware instance mining (MIM) to enhance object instance retrieval. Additionally, memory-aware proposal sampling (MPS) is utilized to balance positive–negative sample learning. The experimental results demonstrate significant gains in the detection accuracy compared to the baselines, showcasing the effectiveness of the approach.

The final set of papers in this Special Issue explores groundbreaking innovations in sensing and imaging technologies. From non-line-of-sight imaging using echolocation to quick-response eigenface analysis schemes for brain–computer interfaces, these studies push the boundaries of what is possible in remote sensing, imaging, and neurotechnology. By introducing novel methodologies and leveraging cutting-edge technologies, these papers pave the way for future advancements in sensing and imaging applications.

The work in [10], entitled “Deep Non-Line-of-Sight Imaging Using Echolocation”, introduces a novel approach to non-line-of-sight (NLOS) imaging using acoustic equipment inspired by echolocation. This paper offers a promising alternative to optical NLOS imaging. Unlike optical systems, which rely on diffused light, our method leverages echoes to visualize hidden scenes. Traditional acoustic NLOS methods suffer from noise and long acquisition times. To address this, the authors propose simultaneous echo collection and deep learning models to overcome interference challenges. The model successfully reconstructs hidden object outlines, offering a promising alternative to optical NLOS imaging.

The work in [11], entitled “A Novel Quick-Response Eigenface Analysis Scheme for Brain–Computer Interfaces”, introduces a novel quick-response eigenface analysis (QR-EFA) scheme for motor imagery in brain–computer interfaces (BCIs). Leveraging EEG signals in a standardized QR image domain, they combine EFA with a convolutional neural network (CNN) for neuro image classification. To address non-stationary BCI data and non-ergodic characteristics, they employ effective neuro data augmentation during training. QR-EFA enhances the classification accuracy by maximizing similarities in domain, trial, and subject directions. QR-EFA enhances the classification accuracy by maximizing similarities in domain, trial, and subject directions. The experimental results on BCI competition datasets demonstrate significant performance improvement over previous methods, achieving classification accuracies of 97.87%.

Lastly, the authors of [12] aim to utilize technology to develop systems capable of recognizing Arabic Sign Language (ArSL) through deep learning techniques. They propose a hybrid model designed to capture the spatio-temporal aspects of sign language, including both letters and words. This hybrid model incorporates a convolutional neural network (CNN) to extract spatial features from sign language data and a Long Short-Term Memory (LSTM) network to capture spatial and temporal characteristics for handling sequential data, such as hand movements.

In conclusion, the diverse range of papers presented in this editorial overview highlights the interdisciplinary nature of research in deep learning-based imaging and sensing. These studies not only showcase the latest advancements in this area of research but also provide valuable insights and methodologies that have the potential to impact various fields, from healthcare and astronomy to robotics and beyond.
